# The relationship between physical exercise and subjective well-being among Chinese junior high school students: A chain mediating model

**DOI:** 10.3389/fpsyg.2022.1053252

**Published:** 2023-01-09

**Authors:** Shu-Jun Yao, Qi-Shuai Ma, Chao Liu, Da-Wei Cao, Teng Lyu, Ke-Lei Guo

**Affiliations:** ^1^Department of Physical Education, Guang Dong Technology College, Zhaoqing, China; ^2^School of Physical Education, Huaibei Normal University, Huaibei, China; ^3^School of Economic and Management, Shanghai University of Sport, Shanghai, China; ^4^Graduate School, University of Perpetual Help System Dalta, Manila, Philippines; ^5^School of Physical Education and Health, Zhaoqing University, Zhaoqing, China

**Keywords:** physical exercise, perceived social support, physical exercise self-efficacy, subjective well-being, junior high school students

## Abstract

**Objective:**

This study aims to help understand the mechanism behind the relationship between physical exercise and the subjective well-being among Chinese junior high school students, and it is of great significance for the intervention measures to improve the subjective well-being of junior high school students.

**Methods:**

Using stratified cluster sampling method, 1,510 junior high school students (727 males and 783 females) were measured by physical exercise rating scale, perceived social support scale, physical exercise self-efficacy scale, and subjective well-being scale. For data analysis, Pearson’s correlation analysis, structural equation model test, and bias-corrected percentile Bootstrap method were carried out in turn.

**Results:**

Common method biases can be accepted in this study. (1) There is a positive correlation between physical exercise and subjective well-being, and physical exercise has a significant predictive effect on subjective well-being(*β* = 0.367 *t* = 9.415 *p* < 0.01); (2) Perceived social support partially mediated the relationship between physical exercise and subjective well-being (*β* = 0.08, *t* = 3.083, *p* < 0.01), and its effect ratio is 78.047%; (3) Physical exercise self-efficacy plays a partial mediating role between physical exercise and subjective well-being(*β* = 0.181, *t* = 5.132, *p* < 0.01), accounting for 50.632%; (4) The chain mediating effect of perceived social support and physical exercise self-efficacy was significant (the mediating effect value was 0.028), and the effect amount was 7.629%.

**Conclusion:**

(1) Physical exercise can significantly positively predict the subjective well-being of junior high school students; (2) Physical exercise can also indirectly affect the subjective well-being of junior high school students through the mediating effect of perceived social support and physical exercise self-efficacy. The intermediary effect includes three paths, namely, the individual intermediary effect of perceived social support and physical exercise self-efficacy and the chain intermediary effect of perceived social support physical exercise self-efficacy.

## Introduction

From the perspective of positive psychology, subjective well-being is an important content reflecting psychological development (Ryff, 1995). It can not only evaluate a person’s quality of life, but also measure a person’s mental health and psychological development level (Chen, 2004).

China’s secondary school entrance examination system is a junior high school academic level examination based on nine-year compulsory education. The most important thing is the selective examination that students face for the first time. In recent years, Chinese junior high school students are not only facing increasing pressure on their studies, but also the pressure from their families, schools and society. It is easy for junior high school students to have psychological problems such as depression and anxiety, and bear enormous psychological pressure (Guo et al., 2022). This paper studies the relationship between physical exercise and subjective well-being of junior high school students, trying to sort out the influence mechanism of junior high school students’ physical exercise on their subjective well-being.

### Physical exercise and subjective well-being

Physical exercise is a kind of physical activity aimed at strengthening physical condition, adjusting spirit and enriching cultural life by using various physical exercise methods and combining natural forces and health measures (Hong Kong Sports Institute, 2000). Subjective well-being (SWB) is an overall evaluation of an individual’s quality of life according to self-defined criteria, and is also an important indicator to measure a person’s mental health (Diener et al., 1999). In addition to improving physical fitness, physical exercise also has a spillover effect (Hu and Yu, 2019). Researchers generally believe that physical exercise can improve individual subjective well-being (Schnohr et al., 2005). Ji et al. (1998) believe that appropriate physical exercise can make individuals obtain more sports pleasure, and the “smooth” (smooth, intoxicated and peak) experience gained by participants in the process of sports can make people forget those things that undermine our happiness, thus promoting the rise of the happiness base line (Qiao and Fan, 2020). Chen et al. (2013) research team also confirmed that the emotional effects of joy, fluency and peak experienced by participating in physical exercise can directly improve the level of subjective well-being of participants. Diener (2000) believes that the sources of subjective well-being experience include not only shopping, party and chat, but also the happiness gained by participating in physical exercise, which can not be replaced by other sources. Claus’s (2017) research shows that by using the gradual relaxation exercise method, and by intervening individual anxiety, the research on the influence of physical exercise on subjective well-being has confirmed that physical exercise can make people obtain a durable and relatively stable state of well-being. The results of the research on the relationship between physical exercise and subjective well-being are mainly as follows: correlation and causality. Scholars adopted the method of stratified random sampling survey (Zang, 2009). Through the survey, it was found that there was a significant positive correlation between the level of physical exercise and the level of subjective well-being, that is, the level of subjective well-being of students who regularly participated in physical exercise was higher than that of students who did not participate in physical exercise (Zhang, 2003). Based on this, we propose the following hypotheses:

*Hypothesis 1*: Physical exercise can positively predict subjective well-being. (H1).

### Intermediary role of perceived social support

Perceived social support is a psychological fact, which contains people’s perception of the social support they have received, and represents the individual’s expectation and understanding ability of the social support they have (Dunkel-Schetter and Bennett, 1990). Perceived social support is often regarded as a mediating variable in addition to its direct effect on other psychological variables. For example, in Ye et al.’s (2012) study on the relationship between feelings, perceived social support and positive attribution style, perceived social support has no direct effect on feelings, but plays a mediating effect. Yang and Ye (2014) studied the subjective well-being of adolescent students and confirmed that perceived social support can be used as a mediating variable to affect adolescent students’ well-being. Some studies have also shown that perceived social support has a mediating effect on the relationship between physical exercise and subjective well-being. For example, the study of Dong et al. (2019) showed that when individuals participated in physical exercise and received support from family and friends, they could generate lasting enthusiasm and interest in physical exercise. When they experience more social support, they will have a more positive description of themselves, and thus a higher self-evaluation, thus improving the level of subjective well-being. Panza et al. (2019) investigated the relationship between physical exercise habits and subjective well-being. The study found that if physical exercise and subjective well-being is low, physical exercise can not directly affect subjective well-being, but by social support and sense of self health such as intermediary variable indirect effect on subjective well-being, individual comprehend from friends and family support, will have great influence on the level of subjective well-being (Xing, 2005). Lian (2009) confirms this view. Yang and Ye (2014) investigated the subjective well-being of adolescents and confirmed that perceived social support, as a mediating variable, affects the well-being of adolescent students. According to the research, when individuals experience more social support, they will have a more positive description of themselves, which will lead to positive self-evaluation, increase social activities, and improve their subjective well-being level (Xiong, 2008). Thus, we propose the following hypotheses:

*Hypothesis 2*: Perceived social support plays a mediating role between physical exercise and subjective well-being. (H2).

### Mediating effect of physical exercise self-efficacy

Another mediating variable of interest in this study is physical exercise self-efficacy. Physical exercise self-efficacy refers to the belief that individuals actively participate in and adhere to a certain intensity and duration of physical exercise ability (Motl et al., 2000). Some studies have found that self-efficacy acts as a mediator between variables and subjective well-being (Meng et al., 2010). With the deepening of cross-theoretical research, in the study of the effect mechanism of physical exercise self-efficacy. Wang et al. (2016) found that physical exercise self-efficacy partially mediated the study of group leadership behavior, team cohesion and exercise persistence. Yan et al.’s (2019) research on the relationship between transactional leadership behavior and willingness to persist in exercise of college P.E. teachers shows that self-efficacy of physical exercise plays a mediating role. Liu et al. (2017) also found that self-esteem and physical exercise self-efficacy had a statistically significant chain mediating effect between physical exercise and life satisfaction. Liu and Ma (2017) conducted a study on middle school students in boarding schools of *Tacheng* farming and pastoral areas, and confirmed that physical exercise was related to self-efficacy and subjective well-being. Meanwhile, social cognitive theory holds that self-efficacy can not only influence individual behavior, but also be influenced by individual successful behavior. Physical exercise strengthens the individual’s sense of ability and value, thus enhancing subjective well-being (Barr-Anderson et al., 2007). Thus, we propose the following hypotheses:

*Hypothesis 3*: Physical exercise self-efficacy plays a mediating role between physical exercise and subjective well-being. (H3).

### Chain mediating effect of perceived social support and physical exercise self-efficacy

Perceived social support has a significant effect on physical exercise self-efficacy. For example, Bandura (1987) believed that social pressure could affect people’s sense of efficacy through research. Sebastian (2013) further explained that when individuals receive positive or negative feedback from acquaintances, their self-efficacy changes accordingly depending on the type of feedback they receive. Au et al. (2009) found that self-efficacy and social support were significant predictors of life satisfaction. Therefore, the internal and external factors of perceived social support and physical exercise self-efficacy can jointly affect subjective well-being, which has a potential dual mediating effect. Song et al. (2010) showed that perceived social support can effectively relieve students’ psychological pressure, improve their sense of self-efficacy, help students to face learning with a more positive attitude, and then have a positive impact on students’ subjective well-being. Therefore, perceived social support and physical exercise self-efficacy, as internal and external factors, can jointly influence subjective well-being and have potential dual mediating effects. In conclusion, physical exercise self-efficacy not only plays a mediating effect between variables and subjective well-being, but also affects individual physical exercise self-efficacy through perceived social support, which can further affect individual subjective well-being experience. Thus, we propose the following hypotheses:

*Hypothesis 4*: Perceived social support and physical exercise self-efficacy play the chain mediating roles between physical exercise and subjective well-being. (H4).

**Figure 1:**
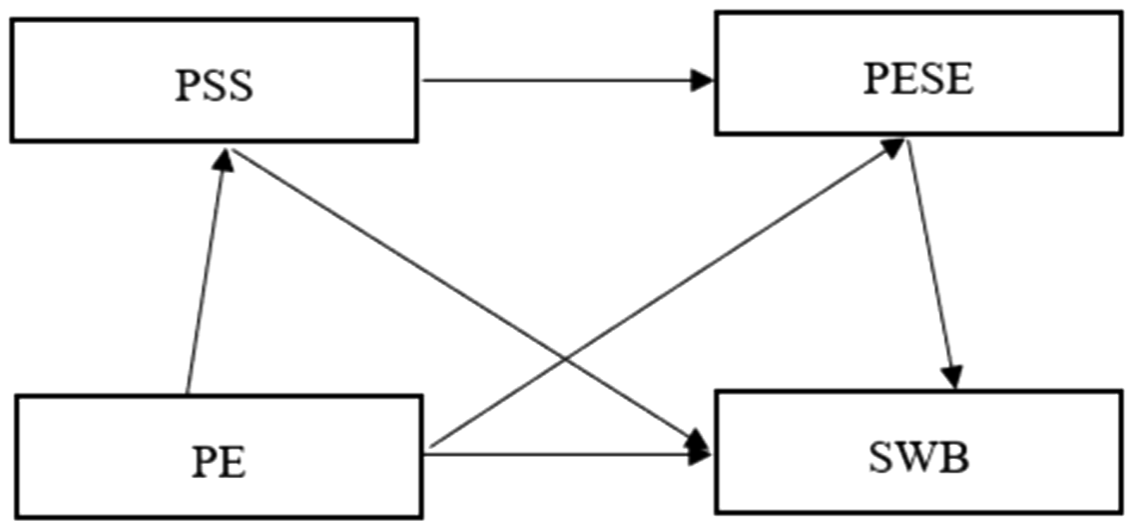
Conceptual model. PE, Physical Exercise; PSS, Perceived Social Support; PESE, Physical Exercise Self-Efficacy; SWB, Subjective Well-Being.

To sum up, we propose the following four hypotheses and build a conceptual model (see Figure 1).

*H1*: Physical exercise can positively predict subjective well-being.

*H2*: Perceived social support plays a mediating role between physical exercise and subjective well-being.

*H3*: Physical exercise self-efficacy plays a mediating role between physical exercise and subjective well-being.

*H4*: Perceived social support and physical exercise self-efficacy play the chain mediating roles between physical exercise and subjective well-being.

## Materials and methods

### Procedure and participants

**Figure 2:**
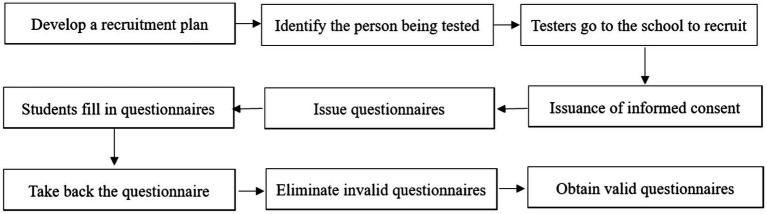
Recruiting flowchart.

A cross-sectional survey was conducted by using the convenience sampling method in Shandong Province. In order to make the sample more representative, economic level and other reasons are considered. This study randomly selected one junior high school in urban and rural areas in the western, central and eastern parts of Shandong Province (6 junior high schools in total). Two classes will be randomly selected from each grade of each junior high school (36 classes in total), and 1,600 questionnaires will be issued. The recruitment flow chart is shown in Figure 2. The students were tested in the classroom, and the main testers were all psychology students who had received professional training. The test was approved by the school leaders, head teachers and participants, and all questionnaire were completed within 10 min. After the questionnaire was collected, follow the following criteria for exclusion: (1) missing date; (2) respond regularly; (3) inconsistent answers, and 1,510 valid questionnaires were recovered with a recovery rate of 94.38%. The participants including 727 boys and 783 girls. There are 509 students in Grade one, 541 students in Grade two, 460 students in Grade three.

### Demographic characteristics of the study sample

As shown in Table 1, of the total sample, 727 were boys, and 783 were girls. The PE level of boys was significantly higher than that of girls, PSS level of boys was significantly higher than that of girls, PESE level of boys was significantly higher than that of girls, SWB level of boys was significantly higher than that of girls.

**Table 1:** Differences in gender.

Variable	Gender	Number	*M*	SD	*t*	*p*
PE	Male	727	24.366	9.991	6.798	<0.001
	Female	783	14.345	6.134		
PSS	Male	727	5.344	1.124	4.97	<0.001
	Female	783	5.635	1.152		
PESE	Male	727	2.272	0.480	6.227	<0.001
	Female	783	2.113	0.515		
SWB	Male	727	11.107	2.420	5.786	<0.001
	Female	783	10.400	2.320		

PE, Physical Exercise; PSS, Perceived Social Support; PESE, Physical Exercise Self-Efficacy; SWB, Subjective Well-Being.

### Measures and instruments

#### Physical exercise

The physical exercise was measured by *Physical Activity Rating Scale* (PARS-3), and it compiled by Liang (1994). The study of Qi (2014) and Fu and Fan (2016) used *PARS-3* to measure physical exercise among Chinese junior students. The scale includes three items, which are, respectively, studied by the intensity of physical exercise, the time of each exercise and the frequency of monthly exercise, so as to measure the level of participation in physical exercise. Each aspect of the scale is divided into 5 levels, and the scale is scored on a 5-level scale, that is, the intensity, time and frequency are graded from 1 to 5 and scored 1–5, respectively. Score of physical exercise amount = score of exercise intensity × (score of exercise time − 1) × score of exercise frequency. The highest score for physical activity is 100, and the lowest is 0. According to the division rules of “Physical Exercise Rating Scale,” the total score equal to or less than 19 is considered as small physical exercise, the score of 19–43 (excluding 19 and 43) is considered as medium physical exercise, and the score equal to or more than 43 is considered as large physical exercise. The higher the score means the higher the individual physical exercise. Based on previous experience (Xia et al., 2018), this study divided the small amount of physical exercise into two parts: no physical exercise and small amount of physical exercise. Therefore, the amount of physical exercise in this study was divided into four grades: no physical exercise, small physical exercise, medium physical exercise and large physical exercise. PARS-3 had reasonable internal consistency coefficient (*α* = 0.639) and test–retest reliability (*r* = 0.82) in previous studies (Xia et al., 2018). In this study, the Cronbach *α* of *PARS-3* was 0.75.

#### Perceived social support

The perceived social support was measured by *Perceived Social Support Scale* (PSSS), and it compiled by Yan and Zheng (2006). In previous studies (Zhuang et al., 2016; Zhang et al., 2020), PSSS was used to measured perceived social support among Chinese junior students and it had good reliability and validity. The *PSSS* is a 7-point-Likert scale and comprises 12 items. It has 3 dimensions: family support (e.g., “I can talk about my problems with my family”), friends support (e.g., “My friends can share happiness and sadness with me”) and other support (teachers, classmates and relatives; e.g., “In my life, teachers, classmates and relatives care about my feelings.”). Each item is valued from 1 (completely disagree) to 7 (completely agree). The score is represented by the average score, and a higher score means higher level of the perceived social support (Zhuang et al., 2016). In the present study, confirmatory factor analysis results demonstrate that date fitting very well: *χ*^2^*/*df = 4.835, RMSEA = 0.078, GFI = 0.935, CFI =0.922, and the Cronbach’s *α* was 0.90.

#### Physical exercise self-efficacy

Physical exercise self-efficacy was measured by Chinese version of *Physical Exercise Self-Efficacy Scale* (PESES; Wu et al., 2002). In previous studies, the *PESES* showed good validity and reliability among Chinese junior students (Zheng, 2019; Dong et al., 2020). The *PESES* is a 3-point-Likert scale and comprises 12 items (e.g., “Even if I have to get up early on weekends, I will keep exercising”). Each item is valued from 1 (I cannot do it) to 3 (I’m sure I can do it). The score is represented by the average score, and a higher score means higher level of the Physical exercise self-efficacy (Luo et al., 2019). In the present study, confirmatory factor analysis results demonstrate that date fitting very well: *χ*^2^*/*df = 4.495, RMSEA = 0.061, GFI = 0.965, CFI =0.952, and the Cronbach’s *α* was 0.87.

#### Subjective well-being

*Index of Well-Being Scale* was compiled by Campbell (1976). Mei et al. (2015) translated and revised this scale to measure the subjective well-being among Chinese junior students. This study used Chinese version of *Index of Well-Being Scale* to measure subjective well-being. *Index of Well-Being Scale* comprises 9 items and divided into two indicators: overall emotional index (8 items; e.g., “How do you feel about life recently, scored from boring to interesting”) and life satisfaction index (1 item; e.g., “How satisfied are you with life in general”). Each item is scored from 1 to 7 points, and the total score is the mean score of the overall emotional index plus the life satisfaction index score and can be from 0 to 14, a higher score shows a higher level of individual happiness (Anglim et al., 2020). In previous studies, *Index of Well-Being Scale* has a fair retest reliability (*r* = 0.91; Zhou et al., 2020). In the present study, confirmatory factor analysis results demonstrate that date fitting very well: *χ*^2^*/*df = 1.40, RMSEA = 0.036, GFI = 0.974, CFI = 0.991, and the Cronbach’s *α* was 0.88.

### Statistical analysis

Invalid data removed after questionnaire recall, and statistical analysis was carried out using the socio-statistical analysis software SPSS 23.0 and the Process plug-in. Firstly, testing for common method bias using Common latent factor. Secondly, SPSS 23.0 was used to examine the Pearson bivariate relationships between physical activity, perceived social support, physical activity self-efficacy and subjective well-being among junior high school students. Thirdly, using the PROCESS plug-in model4 to examine the separate mediating roles of perceived social support and physical exercise self-efficacy. Fourthly, using the PROCESS plug-in model6 and Bootstrap method test the chain mediating effect of perceived social support and physical exercise self-efficacy. In this study, *p* < 0.05 is reasonable.

## Results and analysis

### Common method bias test

Common method bias refers to artifactual covariation between a predictor and a valid scale variable because of the same data source or rater, the same measurement environment, the context of the item, and the characteristics of the item itself. Because this study collected data through self-reporting methods, it was possible that there could be an issue with common method variance (CMV). To reduce this possible deviation, according to the suggestion by Zhou and Long (2004) in the data collection stage, the participants were told that the results would be kept anonymous. It is stated that the data are for scientific purposes only, in order to control for sources of common method bias as much as possible. In order to further improve the rigor of the study, Harman’s one-factor test was used for statistical control before data analysis, that is, unrotated principal component factor analysis is performed on all variables. The results show that the fit between the data and the model is not high (*χ^2^/*df = 40.12, CFI = 0.54, NNFI = 0.58, NFI = 0.63, RMESA = 0.33). There is no obvious common methodological bias in this study.

### Descriptive statistics and correlation analysis

*Pearson* bivariate correlation analysis results of each variable are shown in Table 2. Physical exercise was positively correlated with subjective well-being, physical exercise self-efficacy and perceived social support. Physical exercise self-efficacy and perceived social support were positively correlated with subjective well-being. Physical exercise self-efficacy was positively correlated with perceived social support.

**Table 2:** Mean, standard deviation, and correlation coefficient of each variable.

	*M*	SD	1	2	3	4
1. PE	19.161	8.455	1			
2. SWB	5.331	1.188	0.236^**^	1		
3. PESE	2.193	0.505	0.248^**^	0.510^**^	1	
4. PSS	5.484	1.147	0.242^**^	0.773^**^	0.501^**^	1

PE, Physical Exercise; PSS, Perceived Social Support; PESE, Physical Exercise Self-Efficacy; SWB, Subjective Well-Being. **p < 0.01.

### The mediating effect of perceived social support between physical exercise and subjective well-being

The mediating effect of perceived social support between physical exercise and subjective well-being was analyzed by the sequential test method in the mediating effect test procedure proposed by Wen and Ye (2014).

Table 3 shows that physical exercise has a significant direct predictive effect on SWB (*t* = 9.415, *p* < 0.01). Physical exercise had a significant direct predictive effect on perceived social support (*t* = 9.672, *p* < 0.01). Hypothesis 1 is verified.

**Table 3:** Analysis results of mediating effect of perceived social support.

	SWB			PSS			SWB		
	*β*	SE	*t*	*β*	SE	*t*	*β*	SE	*t*
Constant	4.250^**^	0.119	35.859	4.414^**^	0.114	38.631	0.772^**^	0.109	7.095
PE	0.367^**^	0.039	9.415	0.363^**^	0.038	9.672	0.080^**^	0.026	3.083
PSS							0.788^**^	0.017	45.356
*R* ^2^	0.056			0.058			0.601		
Δ*R*^2^	0.055			0.058			0.6		
*F*	*F* (1,1,508) = 88.639, *p* = 0.000			*F* (1,1,508) = 93.546, *p* = 0.000			*F* (2,1,507) = 1133.320, *p* = 0.000		

PE, Physical Exercise; PSS, Perceived Social Support; SWB, Subjective Well-Being.

^**^p < 0.01.

When physical exercise and perceived social support were added to the regression equation, the predictive effect of physical exercise on SWB was still significant (*t* = 3.083, *p* < 0.01). Perceived social support also positively predicted SWB (*t* = 45.356, *p* < 0.01). Formula A * B/C was calculated according to the proportion of effects. Perceived social support partially mediated the relationship between physical exercise and subjective well-being. The effect accounted for 78.047%. Hypothesis 2 is verified.

### Mediating effect analysis of physical exercise self-efficacy between physical exercise and subjective well-being

It can be seen from Table 4. Physical exercise had a significant direct predictive effect on SWB (*t* = 9.415, *p* < 0.01). Physical exercise had a significant direct predictive effect on physical exercise self-efficacy (*t* = 9.929, *p* < 0.01). When physical exercise and physical exercise self-efficacy were added to the regression equation. The predictive effect of physical exercise on SWB was still significant (*t* = 5.132, *p* < 0.01). Physical exercise self-efficacy also positively predicted subjective well-being (*t* = 21.242, *p* < 0.01). Physical exercise self-efficacy partially mediates the relationship between physical exercise and subjective well-being. The effect accounted for 50.623%. Hypothesis 3 is verified.

**Table 4:** Analysis results of mediating effect of physical exercise self-efficacy.

	SWB			PESE			SWB		
	*β*	SE	*t*	*β*	SE	*t*	*β*	SE	*t*
Constant	4.250**	0.119	35.859	1.707**	0.05	34.003	2.315**	0.138	16.749
PE	0.367**	0.039	9.415	0.164**	0.016	9.929	0.181**	0.035	5.132
PESE							1.133**	0.053	21.242
*R* ^2^	0.056			0.061			0.273		
*Δ*R^2^	0.055			0.061			0.272		
*F*	*F* (1,1,508) = 88.639,p = 0.000			*F* (1,1,508) = 98.586,p = 0.000			*F* (2,1,507) = 283.151,p = 0.000		

PE, Physical Exercise; PESE, Physical Exercise Self-Efficacy; SWB, Subjective Well-Being. **p < 0.01.

### The chain mediating effect analysis of perceived social support and physical exercise self-efficacy between physical exercise and subjective well-being

The bias corrected Bootstrap method was used to test the chain mediating effect of perceived social support and physical exercise self-efficacy between physical exercise and subjective well-being (Fang and Zhang, 2012). 5,000 Bootstrap samples were randomly selected from the original sample to estimate indirect effects.

**Figure 3:**
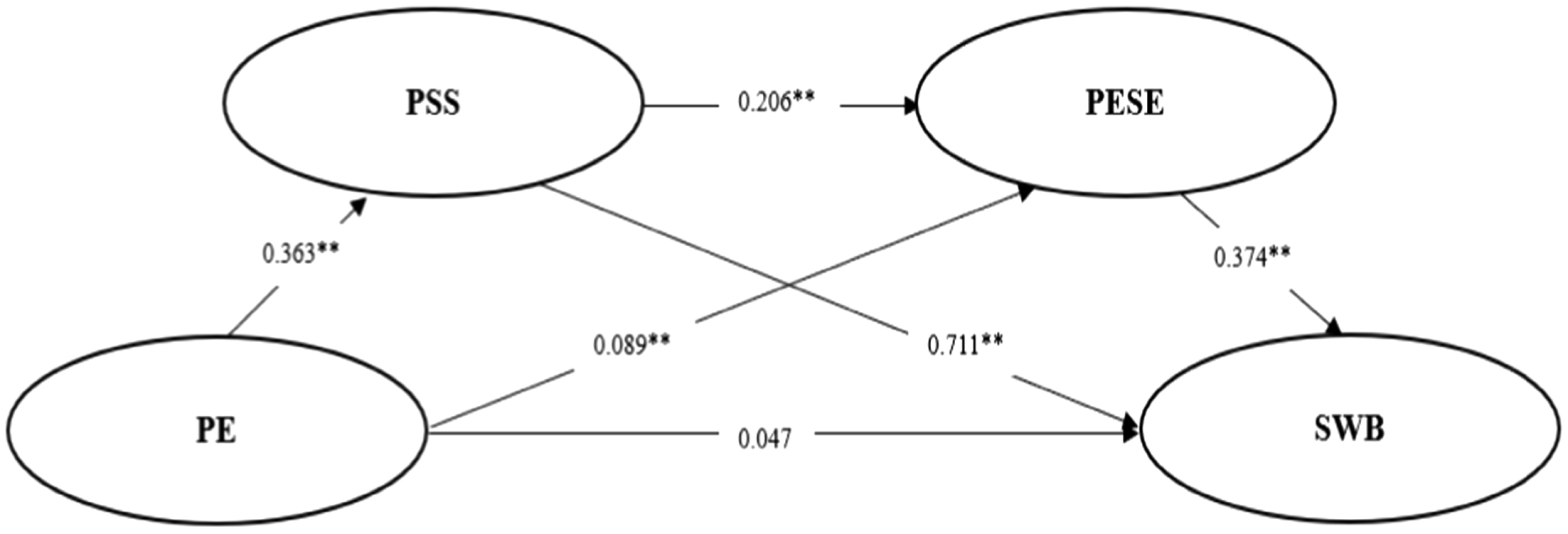
Chain mediation model of physical exercise and subjective well-being. PE, Physical Exercise; PSS, Perceived Social Support; PESE, Physical Exercise Self-Efficacy; SWB, Subjective Well-Being. **p < 0.01.

Table 5 shows the 95% confidence intervals of the Bootstrap sampling test for each path. If the 95% interval does not include the number 0, the mediating effect is significant. According to Table 2. The 95% confidence intervals of the three influence paths do not contain the number 0. The mediating effect of perceived social support was significant (the mediating effect value was 0.258). The effect size was 70.299%; The mediating effect of physical exercise self-efficacy was significant (the mediating effect value was 0.033). The effect size was 8.991%. The chain mediating effect of perceived social support and physical exercise self-efficacy was significant (the mediating effect value was 0.028). The effect size was 7.629%. Hypothesis 4 is verified. The model diagram is shown in Figure 3.

**Table 5:** Bootstrap analysis of significance test of mediating effect.

Affect the path	Effect	Boot SE	Boot LLCI	Boot ULCI
PE ⇒ PSS ⇒ SWB	0.258	0.019	0.132	0.209
PE ⇒ PESE ⇒ SWB	0.033	0.003	0.013	0.026
PE ⇒ PSS ⇒ PESE ⇒ SWB	0.028	0.002	0.013	0.021

PE, Physical Exercise; PSS, Perceived Social Support; PESE, Physical Exercise Self-Efficacy; SWB, Subjective Well-Being.

## Discussion

### The relationship between physical exercise and subjective well-being

It was found that physical exercise, perceived social support and self-efficacy of physical exercise were significantly positive predictors of SWB. Adolescent students are at a critical time in their lives, both physically and mentally. The structure, function and psychological development of their body organs have great plasticity and development. Influenced by “exam-oriented education,” China’s basic education still focuses on academic goals. It neglects the problems in the physical and mental health of young students (Ding and Wang, 2004), which is extremely unfavorable to the healthy growth of young students. Therefore, it is of great practical significance to enhance the physical exercise and subjective well-being of young students to promote their psychological development and healthy growth. The results showed that physical exercise positively predicted the subjective well-being of junior high school students. The more junior high school students participate in physical exercise, the higher their subjective well-being level. This finding is backed up by previous studies (Chen and Ji, 2006). When regular physical exercise takes place over a long period of time, individuals regularly experience pleasurable emotions and form habits. If 1 day does not complete the physical exercise task, the body will have the uncomfortable reaction. Physical exercise activity can affect the physical and emotional health of junior high school students in early development. Therefore, junior high school students can promote the rise of subjective well-being baseline by participating in physical exercise. Lay the foundation for a lifetime of happiness (Lee et al., 2020).

### Understanding the mediating effect of perceived social support and physical exercise self-efficacy

This study not only explored the direct relationship between physical exercise and subjective well-being, but also constructed a chain mediating model through the mediating roles of perceived social support and physical exercise self-efficacy between them and further discusses how physical exercise affects the subjective well-being of junior middle school students. The research results showed that the chain mediating effect of perceived social support and exercise self-efficacy was significant, indicating the importance of mediating variables in explaining physical activity to enhance the subjective well-being of junior high school students.

In this study, perceived social support was significantly positively correlated with subjective well-being of junior high school students, it is consistent with the research results of Li et al. (2017) and Wang and Zhang (2015). Research on perceived social support and well-being has shown that the more social support a person receives, the stronger his subjective well-being will be. High levels perceived social support can lead to a higher level of self-harmony, which can improve their subjective well-being. (Jiang et al., 2013). Social support theory proposed that the stronger the social support network, the stronger the individual’s ability to cope with various challenges (Jiang and Li, 2013). More importantly, this study found that perceived social support partially mediated the relationship between physical exercise and subjective well-being of junior high school students. This means that physical exercise can directly affect junior high school students’ subjective well-being on the one hand, and indirectly affect junior high school students’ subjective well-being through perceived social support on the other hand, this may be due to the fact that team sports provide junior high school students with opportunities to interact with peers and thus help them gain support (Jia, 2021). At the same time, when individuals encounter difficulties, the more they perceive the support from society, family and friends, the more they can solve problems in a positive way and avoid risks. Teachers and classmates are the closest people to students in school. When students perceive support from teachers or peers. Their subjective well-being also increases.

In addition, physical exercise self-efficacy could positively predict the subjective well-being of junior high school students. With the deepening of cross-theoretical research. In the study on the mechanism of physical exercise self-efficacy. Zhang and Wang (2015) found that individuals who regularly participate in physical exercise are good at communicating with others. When encountering negative stimulation will take a variety of effective measures to relieve anxiety. Therefore, negative emotional experience is less. Easy to produce happiness. This study is consistent with the results of Yuan and Zhang (2015). Physical exercise indirectly affects the subjective well-being of junior middle school students through physical exercise self-efficacy. Either after a single strenuous physical exercise or long-term exercise intervention can have a positive impact on physical exercise self-efficacy (Liu, 2020). Secondly, self-efficacy is one of the core contents of Bandura’s social cognitive theory. People with a high sense of self-efficacy are interested in new things and devote themselves to them. Can make constant efforts to overcome difficulties. In this process, their sense of self-efficacy will be constantly strengthened. The self-efficacy of physical exercise will also affect people’s emotions (Guo and Jiang, 2003). This may be due to physical exercise in individuals with high self-efficacy. They tend to be more positive and confident when encountering difficulties or setbacks. A sense of control and competence increases in the process of completing difficulties and challenges. Negative emotional experience is less. Therefore, the level of subjective well-being rises. In addition, the “smooth” experience of physical exercise is also one of the manifestations of positive state traits and can subtly affect subjective well-being (Tan et al., 2020). Junior high school students through long-term regular physical exercise can make their subjective happiness more prominent. Exercise situations with “happiness schema” can improve physical exercise self-efficacy as a fact. The results show that physical exercise self-efficacy is one of the important Bridges connecting physical exercise and mental health. Participating in physical exercise can not only effectively improve the subjective well-being of junior high school students, but also improve the mental health level of junior high school students by improving their physical exercise self-efficacy.

### Physical exercise positively predicts subjective well-being through chain mediation

This study showed that perceived social support and physical exercise self-efficacy had a significant chain mediating effect. The mediation effect value was 0.028. Understanding social support through physical exercise of self-efficacy. The effect size of promoting subjective well-being of junior high school students reached 7.629%. That is, physical exercise can not only directly predict the subjective well-being of junior high school students, but also through understanding the chain effect of social support and physical exercise self-efficacy. It has an indirect effect on the subjective well-being of junior high school students. This is basically consistent with the research results of Au et al. (2009). This may be physical exercise strengthens the individual’s sense of ability and value (Jackson et al., 2021). Relieve individual psychological pressure. Improved self-evaluation of health status (Zhang, 2010). The self-efficacy of individual physical exercise is enhanced. Help them take a more positive attitude toward a game or study, and have a positive impact on subjective well-being. According to social learning theory. The process of interaction between individuals and environment and its results show that the information related to individual interaction belongs to self-efficacy information. It is through cognitive processing of such information that self-efficacy is formed (Chen, 2016). According to Bandura’s theory of four sources of information that affect the formation and change of self-efficacy. Being encouraged to believe in your own effectiveness by the language of others who think you are capable of performing a task. For example, verbal or social persuasion is the persuasive encouragement, advice and suggestions of others. It is one of the four sources of information that influence the formation and change of self-efficacy (Yan, 2008). When an individual has encouragement or persuasion from parents, teachers, classmates or trusted people. It is beneficial for them to participate in physical exercise better and produce a high level of physical exercise self-efficacy. Be able to accept in a positive manner. In the process, the individual experiences a great deal of positive emotion. And these positive emotions lead to happiness (Liang, 2020). This study has important guiding significance for improving the mental health of junior middle school students. Therefore, when thinking about the relationship between physical exercise and subjective well-being of junior high school students, we should pay attention to the important “bridge” role played by perceived social support and physical exercise self-efficacy.

## Proposal

According to the research results, we can promote subjective well-being by improving physical exercise. In daily life, parents should strengthen the educational function of sports, increase family sports activities, build family sports culture, and guide students to participate in physical exercise. Schools should carry out physical education classes according to projects and interests, pay attention to gender differences and grade differences, make teachers’ teaching more targeted, so as to promote junior high school students to actively participate in physical exercise, shape sports spirit, and obtain a sense of happiness. The school cooperates with the extracurricular sports clubs to improve the quality of extracurricular sports activities. The school encourages students to choose their own “sports club,” and students can learn independently according to their hobbies, so as to master sports skills and develop the habit of physical exercise.

Understanding social support is the medium of the relationship between physical exercise and subjective well-being of junior high school students. The scope of activities of junior high school students is mainly between school and family, and the scope of communication is limited. Therefore, family, teachers and classmates have the greatest impact on themselves. Therefore, a social support system including teachers, classmates and parents should be established to help junior high school students develop their subjective well-being. The teacher-student relationship is one of the most important interpersonal relationships in schools. In schools, teachers should hold thematic class meetings to enhance teacher-student exchanges and fully reflect teachers’ caring behavior. Good peer relationship can also promote students’ adaptation to all aspects of learning and life. It is suggested that on the premise of ensuring safety, we should organize classmate gatherings or extracurricular practice in the class on weekends, so that junior high school students can have closer peer relationships and easily feel the power of support around them. Good parent–child relationship can make students feel safe in terms of parental care. It is suggested that parents should pay more attention to the interpersonal needs of boys and guide them to find various ways to obtain support, such as recording life, family group building, etc.

Physical exercise self-efficacy is an important medium between physical exercise and junior high school students’ subjective well-being. Improving junior high school students’ physical exercise self-efficacy is also a channel to improve their subjective well-being. Teachers, parents and peers have the closest relationship with students. Teachers should set an example and actively participate in the big break sports activities to play a leading role. In education and teaching, teachers should fully consider the original physical quality and ability level of students and take care of the individual differences of students. By participating in parent–child sports activities and conducting “emotional exchanges” with students, parents can let children feel the physical and mental experience brought by physical exercise, improve their self-efficacy of physical exercise from the perspective of psychological needs, and promote their subjective well-being.

## Conclusion

(1) Physical exercise positively predicted the subjective well-being of junior high school students. That is to say, students who often participate in physical exercise have a higher level of subjective well-being than students who do not often participate in physical exercise. (2) Physical exercise indirectly affects the subjective well-being of junior high school students through the mediating effect of perceived social support and physical exercise self-efficacy. There are three paths to this mediation. Namely, the separate mediating effect of perceived social support and physical exercise self-efficacy, and the chain mediating effect of perceived social support and physical exercise self-efficacy.

### Limitations and future directions

There are still some areas to be improved in this study: although this study has further proved the relationship between physical exercise and subjective well-being of junior high school students. At the same time, the inner mechanism of physical exercise affecting the mental health of junior middle school students was discussed. However, this study is a cross-sectional study. Predictive results are only explored on the basis of relevant research. In the future, longitudinal follow-up experiments should be used to study the relationship between variables. In addition, this study only considered the mediating effect of perceived social support and physical exercise self-efficacy between physical exercise and subjective well-being of junior high school students. But in reality there may be other mediating variables such as resilience, interpersonal relationships and self-evaluation. Further research is needed. Follow-up research should be based on promoting the development of adolescents’ physical and mental health. Highlight the education concept of “health first.” To further explore the “dose effect” and the influence of related variables on improving the subjective well-being of junior high school students. To improve the core quality of physical education and health, and promote the development of junior middle school students’ mental health.

This study reveals the influence mechanism of physical exercise on the subjective well-being of junior high school students. It has both theoretical and practical significance. In theory, this study further reveals the influence mechanism of physical exercise on the subjective well-being of junior high school students. It has important theoretical value to understand the causes of subjective well-being of junior high school students. Secondly, the psychological mechanism of physical exercise affecting subjective well-being was discussed. Enrich the sports psychology theory. Finally, the Curriculum Standard for Physical Education and Health serves as a national document guiding physical exercise to promote mental health at the macro level. To explore the influence mechanism of physical exercise on subjective well-being of junior high school students. It can promote the education value theory of PE subject and the core accomplishment theory of PE subject in the Curriculum Standard of PE and Health to give full play. In practice, to explore the formation mechanism of junior high school students’ subjective well-being. It is beneficial to relieve the psychological pressure of junior high school students. Prevent mental illness. Cultivate a positive and healthy attitude. Secondly, it is beneficial to improve the level of subjective well-being of junior high school students. Promote the cultivation of core accomplishment of physical education. Finally, it can provide a basis for the government functional departments to formulate relevant policies. At the same time to improve the quality of physical education and teaching to provide support.

## Data availability statement

The original contributions presented in the study are included in the article/supplementary material, further inquiries can be directed to the corresponding authors.

## Ethics statement

This study was conducted in accordance with the Declaration of Helsinki, and it has been approved by the Human Research Ethics Committee of Guang Dong Technology College.

## Author contributions

S-JY, CL, D-WC, and TL: conceptualization. CL, D-WC, TL, and K-LG: methodology, resources, and supervision. S-JY and Q-SM: software, formal analysis, and visualization. Q-SM, D-WC, and CL: validation. S-JY and K-LG: investigation. S-JY: data curation. S-JY, Q-SM, D-WC, and TL: writing—original draft preparation. S-JY, D-WC, and TL: writing—review and editing. D-WC and TL: funding acquisition. All authors contributed to the article and approved the submitted version.

## Funding

This work was supported by the Key Project of “Anhui Provincial University Humanities and Social Sciences (2021SK27),” the Key Project of “Youth Development Research in Anhui Province (AHQFZ2022009),” and the Key Project of “Huaibei Normal University Teaching Research (2021xjxyj021).”

## Conflict of interest

The authors declare that the research was conducted in the absence of any commercial or financial relationships that could be construed as a potential conflict of interest.

## Publisher’s note

All claims expressed in this article are solely those of the authors and do not necessarily represent those of their affiliated organizations, or those of the publisher, the editors and the reviewers. Any product that may be evaluated in this article, or claim that may be made by its manufacturer, is not guaranteed or endorsed by the publisher.
